# Tuning the Red-to-Green-Upconversion Luminescence Intensity Ratio of Na_3_ScF_6_: 20% Yb^3+^, 2% Er^3+^ Particles by Changes in Size

**DOI:** 10.3390/ma16062247

**Published:** 2023-03-10

**Authors:** Yongling Zhang, Xiang Liu, Mingxing Song, Zhengkun Qin

**Affiliations:** 1 School of Chemistry and Pharmaceutical Engineering, Jilin Institute of Chemical Technology, Jilin 132022, China; 2 College of Information & Technology, Jilin Normal University, Siping 136000, China

**Keywords:** Na_3_ScF_6_: 20% Yb^3+^, 2% Er^3+^, upconversion, size, R/G ratio

## Abstract

Na_3_ScF_6_: 20% Yb^3+^, 2% Er^3+^ samples were synthesized with different reaction times and reaction temperatures using the solvothermal method. We carried out a series of tests on Na3ScF6 crystals. The XRD patterns showed that the monoclinic phases of the Na_3_ScF_6_ samples could be synthesized under different reaction conditions, and doping with Yb^3+^ ions and Er^3+^ ions did not change the crystal structures. The SEM images showed that the sizes of the samples gradually increased with reaction time and reaction temperature. The fluorescence spectra showed that the emission peaks of the prepared samples under 980 nm near-infrared (NIR) excitation were centered at 520 nm/543 nm and 654 nm, corresponding to the ^2^H_11/2_/^4^S_3/2_→^4^I_15/2_ and ^4^F_9/2_→^4^I_15/2_ transitions, respectively. With the increasing size of the samples, the emission intensities at 654 nm increased and the luminescence colors changed from green to red; at the same time, the red-to-green luminescence intensity ratios (IR/IG ratios) increased from 0.435 to 15.106—by as much as ~34.7 times. Therefore, this paper provides a scheme for tuning the IR/IG ratios of Na_3_ScF_6_: 20% Yb^3+^, 2% Er^3+^ samples by changing their sizes, making it possible to enhance the intensity of red upconversion, which has great potential for the study of color displays and lighting.

## 1. Introduction

Lanthanide-doped nanomaterials enable the conversion from near-infrared light to visible light due to the anti-Stokes effect when two or more low-energy photons are sequentially absorbed and higher-energy photons are emitted [1,2,3,4]. These nanomaterials have the advantages of long luminescence lifetimes, narrow emission bands, high penetration abilities and chemical stability, making them suitable for potential applications in bioimaging, infrared photothermal therapy, fluorescent biomarkers, anti-counterfeiting, photocatalysis, photodetectors, optoelectronic devices, sensors and other fields [5,6,7,8,9,10,11,12,13,14,15,16,17,18,19]. In particular, red-upconversion-luminescence nanomaterials can be better suited to biological applications. The wavelength range of red light is 620–750 nm, which is more conducive to applications, such as biomarkers, biological probes and the study of biological tissues, due to its strong penetration depth and small light-scattering range compared to green and blue light [5,6,7,20,21,22]. Over the past few years, various methods for synthesizing nanomaterials that produce red-upconversion luminescence have been investigated. Hao Dong et al. increased the red–green luminescence intensity ratio of Er^3+^ by 2 to 100 times by designing a local structure of the luminescence center of the nanocrystal that kept the Yb^3+^/Er^3+^ luminescence center unchanged and by changing the molar ratio of Na^+^/Re^3+^ and F^−^/Re^3+^, and further increased it by 450 times by the epitaxial growth of CaF_2_ shells [23]. Gan Tian et al. obtained NaYF_4_:Yb^3+^/Er^3+^ nanocrystals with pure red-upconversion luminescence by doping Mn^2+^ ions [24]. Sc, as a special rare earth element different from other lanthanide elements, has a unique electronic configuration and a smaller ionic radius (0.83 Å) such that it can more easily produce red-upconversion luminescence, and so people began to turn to Sc^3+^ as a matrix in materials research. Therefore, in recent years there has been an increasing amount of research on NaScF_4_ nanocrystals co-doped with Yb^3+^ and Er^3+^, which can readily produce red-upconversion luminescence [2,5,25,26,27,28,29,30]. Min Pang et al. directly synthesized water-soluble hexagonal NaScF_4_: Yb^3+^/Er^3+^ nanocrystals with strong red-upconversion luminescence by a hydrothermal method; afterwards, an active shell layer containing Yb^3+^ was wrapped around the NaScF_4_: Yb^3+^, Er^3+^ core nanocrystals, which further increased the intensity of the upconversion luminescence by a factor of 6.8 [6]. Mingshun Yang et al. synthesized water-soluble NaScF_4_: Yb^3+^, Er^3+^ nanocrystals with strong red-upconversion luminescence by a hydrothermal method and controlled red–green luminescence intensity ratios by varying the doping concentrations of Yb^3+^ ions, obtaining more intense red-upconversion luminescence at a relatively low doping concentration [8]. Ding et al. synthesized nanorods with orthogonal crystal-phase KSc_2_F_7_: 20% Yb^3+^, 2% Er^3+^, and strong red-upconversion luminescence was observed [31]. Jingning Shan et al. synthesized NaYF_4_: Yb, Er nanoparticles with sizes ranging from 18 nm to 200 nm and studied the effect of size on their luminescence properties. The results showed that the upconversion luminescence intensity of the nanoparticles was proportional to their size [32]. Zhai et al. synthesized NaYF_4_: 20%Yb, 2%Tm nanoparticles with sizes of 9 nm and 20 nm and tested the fluorescence spectra under 980 nm excitation. The results showed that the upconversion luminescence intensities of the nanocrystals of large size were stronger than those of the nanocrystals of small size [33]. However, the effect of the size of Na_3_ScF_6_ nanocrystals on the ratio of red–green luminescence intensities in Yb^3+^-Er^3+^ co-doped systems is rarely reported.

In this article, we synthesized Yb^3+^-Er^3+^ co-doped Na_3_ScF_6_ samples by the solvent thermal method using ethanol, oleic acid and deionized water. We adjusted the sizes of the Na_3_ScF_6_: 20% Yb^3+^, 2% Er^3+^ samples by controlling the reaction times and the reaction temperatures to investigate the effect of size on the luminescence properties of these samples.

## 2. Experimental

### 2.1. Chemicals

All of the chemical medicines were analytical reagents and were used without further purification. Scandium chloride hexahydrate (ScCl_3_·6H_2_O), erbium chloride anhydrous hexahydrate (ErCl_3_·6H_2_O) and oleic acid (OA) were purchased from Alfa Aesar Co., Ltd., Shanghai, China. Ytterbium chloride hexahydrate (YbCl_3_·6H_2_O) was purchased from Shaoyuan Chemical Technology Co., Ltd., Shanghai, China. Potassium fluoride (KF·2H_2_O) was purchased from Tianjin Guangfu Technology Development Co., Ltd., Tianjin, China. Sodium hydroxide (NaOH) and ethyl alcohol (CH_3_CH_2_OH) were purchased from Tianli Chemical Reagent Co., Ltd., Tianjin, China. Cyclohexane (C_6_H_12_) was purchased from Tianjin Kaixin Chemical Industry Co., Ltd., Tianjin, China.

### 2.2. Synthetic Procedures

Na_3_ScF_6_ samples co-doped with Yb^3+^-Er^3+^ were synthesized by the solvothermal method. First, 5 mmol ScCl_3_·6H_2_O, 2 mmol YbCl_3_·6H_2_O and 1 mmol ErCl_3_·6H_2_O were separately dissolved in 10 mL deionized water to form a 0.5 mol L^−1^ ScCl_3_·6H_2_O aqueous solution, a 0.2 mol L^−1^ YbCl_3_·6H_2_O aqueous solution and a 0.1 mol L^−1^ ErCl_3_·6H_2_O aqueous solution for standby application. Next, 20 mL OA, 10 mL ethanol and 2 mL deionized water were added to the beakers in turn. After that, 0.6 g NaOH was dissolved in 2.5 mL deionized water, which was poured into the above mixtures; the mixtures were then stirred for 30 min until dissolution. Then, 800 μL ScCl_3_·6H_2_O aqueous solution, 450 μL YbCl_3_·6H_2_O aqueous solution and 100 μL ErCl_3_·6H_2_O aqueous solution were respectively absorbed by pipette and slowly dropped into the above mixtures successively, followed by stirring for 30 min until dissolution. Following this, 2 mmol KF·2H_2_O (4 times the amount of rare earth ions) was dissolved in 4 mL deionized water and slowly dropped into the above mixed solutions, which were then stirred continuously for 30 min. After the above steps, the mixed solutions were poured into a 50 mL polytetrafluoroethylene (PTFE) lined reactor and the temperature was set to 160 °C and maintained for 4 h. When the reaction time was over and the mixtures had cooled to room temperature, the products were washed several times with cyclohexane and ethanol and centrifuged before, finally, being dried and preserved. Afterwards, the above operations were repeated and the reaction conditions (reaction temperature and reaction time) were changed: 160 °C, 8 h; 180 °C, 4 h; 180 °C, 8 h; 200 °C, 4 h; and 200 °C, 8 h. In this way, Na_3_ScF_6_: 20% Yb^3+^, 2% Er^3+^ samples were obtained.

### 2.3. Characteristics

X-ray diffraction (XRD) patterns: The crystalline phases of the products were measured using a Model Rigaku Ru-200b X-ray powder analyzer produced by the Rigaku Corporation, Japan (λ = 1.5406 Å, scanning range from 10° to 70°). 

Scanning Electron Microscopy (SEM): The morphologies, including size and shape, were measured with a JSM-7500F scanning electron microscope produced by JEOL, Tokyo, Japan. 

Spectroscopic measurements: The spectra of the upconversion particles were measured using a Hitachi F-4500 fluorescence spectrophotometer produced in Japan.

## 3. Results and Discussion

Our experimental results showed the morphologies and fluorescence intensities of the Na_3_ScF_6_: 20% Yb^3+^, 2% Er^3+^ samples. By varying the reaction time and temperature, the sizes of the samples were changed, as were the luminescence colors, and the changes in the sizes of the samples led to changes in their red–green luminescence intensity ratios (I_R_/I_G_ ratios), which has rarely been seen in rare-earth-doped upconversion particles.

**Figure 1:**
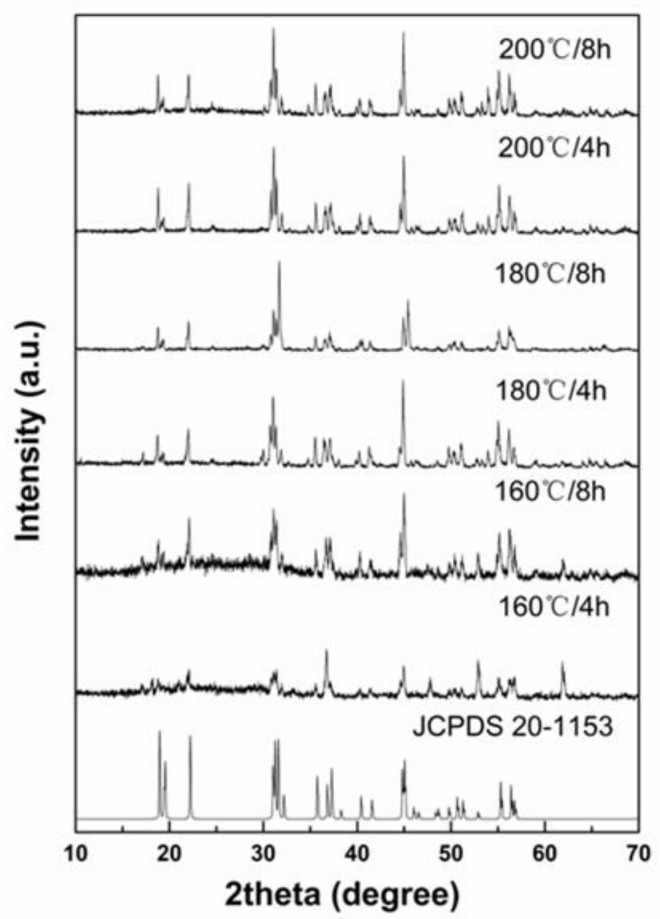
The XRD standard card of Na_3_ScF_6_ (JCPDS-20-1153) and the XRD patterns of Na_3_ScF_6_: 20% Yb^3+^, 2% Er^3+^ samples prepared with different temperatures and reaction times.

The crystal phases of Na_3_ScF_6_: 20% Yb^3+^, 2% Er^3+^ particles prepared under different reaction conditions were analyzed by X-ray diffraction (XRD). The XRD patterns are shown in Figure 1. It can be seen that the diffraction peaks of the prepared samples correspond well to the standard card of the monoclinic-phase Na_3_ScF_6_ (JCPDS card no. 20-1153), and no other impurities could be detected when the temperature was 160 °C and the reaction time was 4 h. When the reaction temperature was constant and the reaction time was increased to 8 h, the diffraction peaks could also be indexed to the standard card of the monoclinic-phase Na_3_ScF_6_. This shows that the samples we prepared were monoclinic-phase Na_3_ScF_6_. With a temperature of 180 °C and a reaction time of 4 h or 8 h, the XRD patterns of the prepared samples corresponded well to the monoclinic-phase Na_3_ScF_6_ (JCPDS card no. 20-1153). The above results show that the prepared samples were pure monoclinic-phase Na_3_ScF_6_. When the temperature reached 200 °C and the reaction time was 4 h or 8 h, similarly, the prepared samples also corresponded to the standard card of monoclinic-phase Na_3_ScF_6_. The above results show that at 200 °C and with a reaction time of 4 h or 8 h, the samples were monoclinic-phase Na_3_ScF_6_. Therefore, it can be seen that when both the temperature and reaction time were varied, the crystal phases of the samples did not change, such that monoclinic-phase Na_3_ScF_6_ could be synthesized, nor did the doping with Yb^3+^ ions and Er^3+^ ions change the crystal structures. However, it can be seen that as the temperature increased and the reaction time increased, the relative peak intensity increased and the diffraction peaks sharpened, indicating that temperature and reaction time can have an effect on the size of samples. The higher the temperature and the longer the reaction time, the higher the degree of the sharpening of the diffraction peaks and the larger the size of the samples. When the reaction temperature was 160 °C and the reaction time was 4 h, the size of the Na_3_ScF_6_ particles was the smallest—only about tens of nanometers—and the shape was spherical; the particles clustered together and they did not form obvious crystals; in other words, the crystallinity was not high and the crystal grains were small, which led to a significant reduction in peak intensity, and some of the peaks even disappeared. 

**Figure 2:**
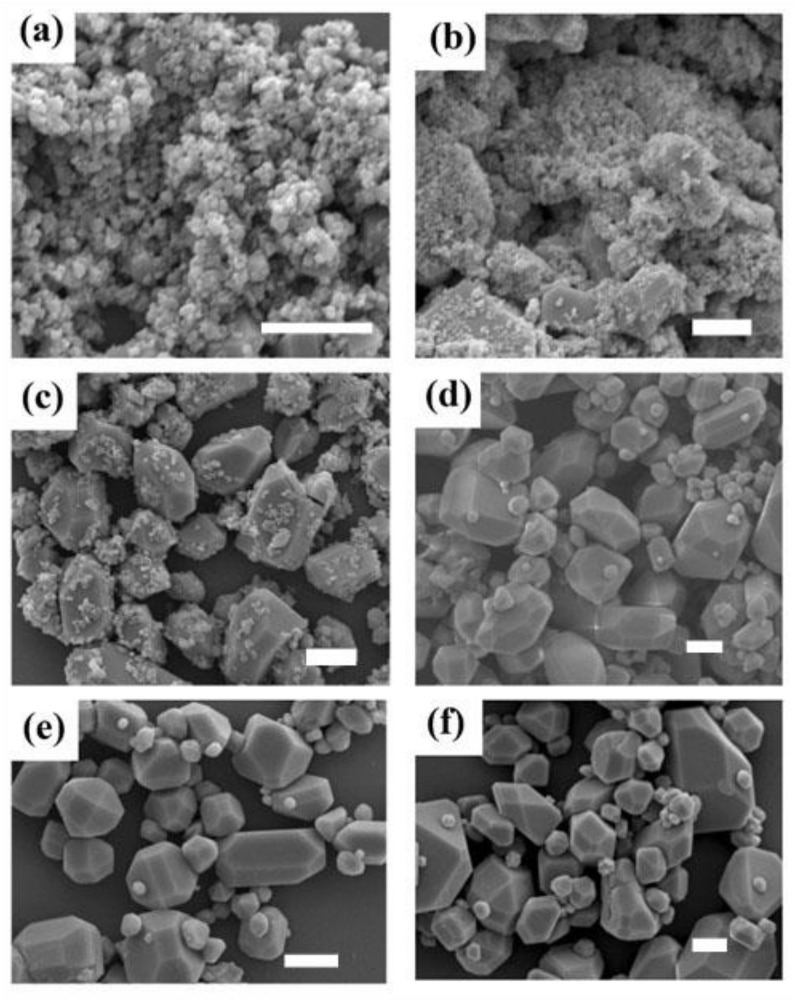
SEM images of Na_3_ScF_6_: 20% Yb^3+^, 2% Er^3+^ particles under different reaction conditions: (**a**) 160 °C, 4 h; (**b**) 160 °C, 8 h; (**c**) 180 °C, 4 h; (**d**) 180 °C, 8 h; (**e**) 200 °C, 4 h; and (**f**) 200 °C, 8 h. The scale bars in the figures represent 1 μm.

Figure 2 shows the SEM images of the Na_3_ScF_6_: 20% Yb^3+^, 2% Er^3+^ particles prepared under different reaction conditions. As shown in Figure 2a, when the reaction temperature was 160 °C and the reaction time was 4 h, the resulting size of the prepared samples was small, the minimum diameter was 28 nm and the maximum diameter reached up to 153 nm; when the temperature was kept constant and the reaction time was increased to 8 h, it could be seen that the size of the prepared samples became larger, with minimum diameters of 61 nm and maximum diameters of 162 nm, as shown in Figure 2b; comparing the results with those obtained with a reaction time of 4 h, the size of the samples increased gradually. It can be seen from Figure 2a,b that the sizes of the samples we prepared were nanometric. As shown in Figure 2c, when the reaction temperature was 180 °C and the reaction time was 4 h, the sample shapes were polyhedral and the sizes of the prepared samples became micrometric. The maximum diameter of the samples was 1.8 μm, and many small particles with an average diameter of about 300 nm were attached to the surfaces. At this temperature, as the reaction time increased to 8 h, the small particles attached to the surfaces gradually grew into small nanocrystals with diameters of about 500 nm; the maximum diameter of the large particles was 2.2 μm, and the surfaces tended to be smooth, as shown in Figure 2d. As shown in Figure 2e, when the temperature was 200 °C and the reaction time was 4 h, the surfaces of the samples were smooth, with an average diameter of 2 μm. At this temperature, the reaction time was increased to 8 h and the size of the nanocrystals was almost the same as the size of the large nanocrystals; the average diameter of the samples was 2.4 μm.

**Figure 3:**
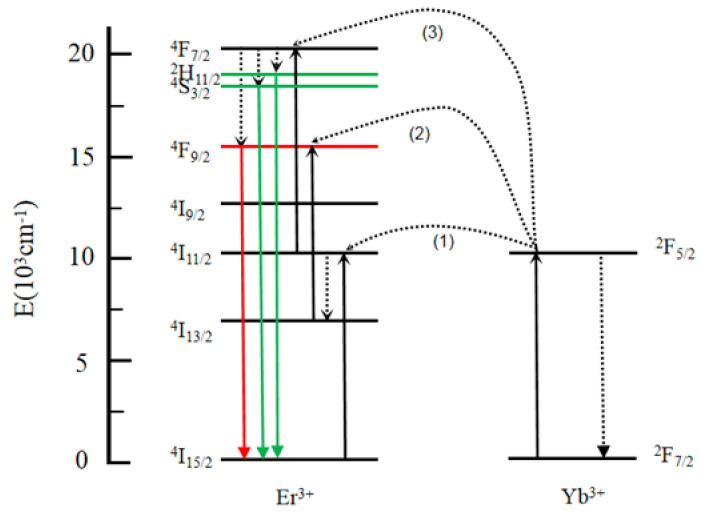
Schematic diagram of the energy-transfer process of the Na_3_ScF_6_: Yb^3+^, Er^3+^ system.

Figure 3 shows the energy-transfer process in the Yb^3+^-Er^3+^ co-doped Na_3_ScF_6_ systems. To the best of our knowledge, the Yb^3+^ ions have a large absorption cross section at 980 nm, which allows the Yb^3+^ ions to continuously absorb photons to be transferred to the adjacent Er^3+^ ions, so we chose Yb^3+^-Er^3+^ co-doped systems as the research objects. We chose Na_3_ScF_6_ as the matrix material because the radius of Sc^3+^ ions is small. In the A-body system, Yb^3+^ ions and Er^3+^ ions take the positions of the Sc^3+^ ions in the lattice. This makes the distance between Yb^3+^ ions and Er^3+^ ions closer, and the energy-transfer efficiency between them is higher. The possible upconversion processes are as follows: (1) Yb^3+^ ions absorb the energy of the 980 nm pump light and the electrons from the ^2^F_7/2_ energy level transition to the ^2^F_5/2_ energy level, and the energy is transferred from Yb^3+^ ions to Er^3+^ ions, the electron layout changing to the ^4^I_11/2_ level of the Er^3+^ ions. (2) The electrons on the ^4^I_11/2_ level of the Er^3+^ ions are arranged at the ^4^I_13/2_ level of the Er^3+^ ions through nonradiative transition. Then, the following energy-transfer process occurs between Yb^3+^ ions and Er^3+^ ions: ^2^F_5/2_(Yb^3+^) + ^4^I_13/2_(Er^3+^) → ^2^F_7/2_(Yb^3+^) + ^4^F_9/2_(Er^3+^). The radiative transition of electrons from the ^4^F_9/2_ level of Er^3+^ ions to the ^4^I_15/2_ level of the ground state of Er^3+^ ions produces red-upconversion luminescence. (3) Due to the close spacing between Yb^3+^ and Er^3+^ ions and the appropriate energy matching between ^4^I_11/2_ → ^4^F_7/2_ (Er^3+^) and ^2^F_7/2_ → ^2^F_5/2_ (Yb^3+^), the energy-transfer process occurs between Er^3+^ and Yb^3+^ ions. The electrons at the ^4^F_7/2_ level of the Er^3+^ ions are arranged at the ^2^H_11/2_, ^4^S_3/2_ and ^4^F_9/2_ level of the Er^3+^ ions through nonradiative transition. The radiative transition of electrons from the ^2^H_11/2_/^4^S_3/2_ level of Er^3+^ ions to the ^4^I_15/2_ level of the ground state of Er^3+^ ions produces green-upconversion luminescence. Simultaneously, the radiative transition of electrons from the ^4^F_9/2_ level of Er^3+^ ions to the ^4^I_15/2_ level of the ground state of Er^3+^ ions produce red-upconversion luminescence. Analytically, we determined that when the reaction time and temperature increased, the size of the Na_3_ScF_6_ nanocrystals increased and the Yb^3+^ ions and Er^3+^ ions that occupied the positions of the Sc^3+^ ions in the Na_3_ScF_6_ nanocrystal systems became closer, leading to an increase in energy-transfer efficiency.

**Figure 4:**
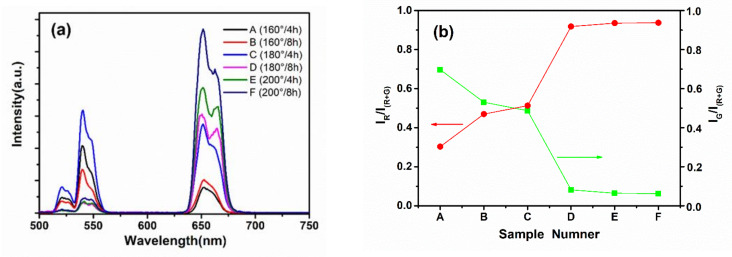
(**a**) Under the excitation of a 980 nm laser, the fluorescence spectra of Na_3_ScF_6_: 20% Yb^3+^, 2% Er^3+^ particles under different reaction conditions. (**b**) The ratio of green emission intensity I_G_ and red emission intensity I_R_ to the total integrated intensity I(_G+R_) of sample A (160 °C, 4 h), sample B (160 °C, 8 h), sample C (180 °C, 4 h), sample D (180 °C, 8 h), sample E (200 °C, 4 h) and sample F (200 °C, 8 h) under different reaction conditions in the range of 500–750 nm.

Figure 4b shows the ratios of green emission intensity I_G_ and red emission intensity I_R_ to the total integrated intensity I_(G+R)_ of sample A (160 °C, 4 h), sample B (160 °C, 8 h), sample C (180 °C, 4 h), sample D (180 °C, 8 h), sample E (200 °C, 4 h) and sample F (200 °C, 8 h) under different reaction conditions in the range of 500–750 nm. It can be seen from Figure 4b that when the reaction temperature was 160 °C and the reaction time was 4 h (sample A), the green emission intensity I_G_ accounted for 70% of the total integrated intensity I_(G+R)_, while the red emission intensity I_R_ accounted for 30%. When the reaction temperature was 160 °C and the reaction time was 8 h (sample B) and when the reaction temperature was 180 °C and the reaction time was 4 h (sample C), the green emission intensity I_G_ was not much different from the red emission intensity I_R_, which was about 50%, but when the reaction temperature was 180 °C and the reaction time was 8 h (sample D) and when the reaction temperature was 200 °C and the reaction time was 4 h (sample E) and when the reaction temperature was 200 °C and the reaction time was 8 h (sample F), the green emission intensity I_G_ only accounted for 5% of the total integrated intensity, while the red emission intensity I_R_ was as high as 95%. It was found that with increases in temperature and time, the ratio of I_G_ in I_(G+R)_ gradually decreased, while IR accounted for I_(G+R)_ gradually increasing, and the color of the corresponding samples changed from green to red. As can be seen in Figure 3a, which shows the fluorescence spectra, the emission peaks were centered at 520 nm/543 nm and 654 nm, corresponding to the ^2^H_11/2_/^4^S_3/2_ → ^4^I_15/2_ (green-upconversion luminescence) and ^4^F_9/2_ → ^4^I_15/2_ (red-upconversion luminescence) transitions, respectively. When the temperature was 160 °C, the size of our samples was at the nanometer level; as the reaction time increased from 4 h to 8 h, the size of the nanocrystals increased slightly and it was found that the green emission decreased and the red emission increased, as can be seen in Figure 3b. When the temperature was 180 °C and the reaction time was 4 h, the samples we prepared had both nanometric and micrometric sizes, the green fluorescence intensity of the samples was weakened and the red fluorescence intensity of the samples was enhanced, compared with the samples prepared when the reaction temperature was 160 °C. When the temperature was 180 °C, as the reaction time increased from 4 h to 8 h, a sharp decrease in green emission at 520 nm/543 nm and a sharp increase in red emission at 654 nm were observed. The above results can be seen in Figure 4b. When the reaction temperature was 200 °C, as the reaction time increased from 4 h to 8 h, the size of the samples increased slightly, and it was found that both had low green emission at 541 nm and slightly higher red emission at 654 nm for the reaction time of 8 h. When the reaction time was kept constant at 4 h or 8 h, the red emission at 654 nm increased gradually as the temperature increased, as was observed in the fluorescence spectra corresponding to the green-luminescence intensity of the samples decreasing gradually and the red-luminescence intensity of the samples increasing gradually. This was due to the small radius of the Sc^3+^ ions, which is only 0.83 Å., making the distance between Sc^3+^-Sc^3+^ in the Na_3_ScF_6_ host shorter, so that when the doped Yb^3+^ ions and Er^3+^ ions enter the host to occupy the position of the Sc^3+^ ions, the distance between the Yb^3+^ ions and Er^3+^ ions is reduced, increasing the probability of cross-relaxation, making it easier for Er^3+^ ions to jump to higher energy levels, such as ^4^F_9/2_; in addition, as the temperature and reaction time increase, the surface defects decrease in the wake of the size of the nanocrystal increase, and the energy-transfer upconversion efficiency is much higher than the surface-defect effect, so the green fluorescence intensity of a sample shows a gradual weakening trend, and the red l fluorescence intensity of a sample shows a gradual weakening trend. 

**Figure 5:**
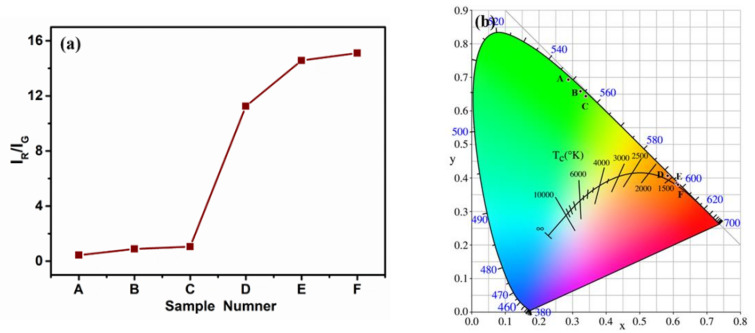
(**a**) The ratio of red emission intensity I_R_ to green emission intensity I_G_ of sample A (160 °C, 4 h), sample B (160 °C, 8 h), sample C (180 °C, 4 h), sample D (180 °C, 8 h), sample E (200 °C, 4 h) and sample F (200 °C, 8 h) under different reaction conditions in the range of 500–750 nm. (**b**) CIE color chromaticity coordinates of Na_3_ScF_6_: 20% Yb^3+^, 2% Er^3+^ particles under different reaction conditions: sample A (160 °C, 4 h), sample B (160 °C, 8 h), sample C (180 °C, 4 h), sample D (180 °C, 8 h), sample E (200 °C, 4 h) and sample F (200 °C, 8 h).

Figure 5a shows the ratios of red emission intensity I_R_ to green emission intensity I_G_ of Na_3_ScF_6_: 20% Yb^3+^, 2% Er^3+^ samples under different reaction conditions in the range of 500–750 nm. Figure 5b shows the CIE color chromaticity coordinates of Na_3_ScF_6_: 20% Yb^3+^, 2% Er^3+^ samples under different reaction conditions. When the temperature was 160 °C and the reaction time was 4 h, the size of the Na_3_ScF_6_: 20% Yb^3+^, 2% Er^3+^ samples was the smallest; at this point in time, the I_R_/I_G_ ratio was about 0.435 (as shown in Figure 5a), and the sample produced green-upconversion luminescence, which could be shown by Figure 5b. When the temperature remained constant and the reaction time increased to 8 h, the size of the Na_3_ScF_6_: 20% Yb^3+^, 2% Er^3+^ samples increased slightly; at this point in time, the I_R_/I_G_ ratio was about 0.885 (as shown in Figure 5a), and from this it can be seen that when the temperature was 160 °C, the size of the samples increased and the I_R_/I_G_ ratio expanded by a factor of ~2 as the reaction time increased. The calculated CIE color coordinates were slightly offset away from the green area, as can be seen in Figure 5b. When the temperature was 180 °C and the reaction time was 4 h, the sample size increased but the surfaces attached to many small particles; at this time, the I_R_/I_G_ ratio was ~1.053 (as shown in Figure 5a) and the calculated CIE color coordinates were further shifted away from the green area (as shown in Figure 5b). When the temperature was 180 °C and the reaction time was 8 h, the size of the samples increased, and the diameters of the small particles attached to the surfaces also increased; at this point, it was clear that the I_R_/I_G_ ratio was ~11.248. Therefore, we found that when the temperature was 180 °C, with an increase in reaction time, the size of the samples increased and the I_R_/I_G_ ratio expanded ~25 times, compared with 160 °C reaction temperature and the 4 h reaction time. It also can be seen from Figure 5a that when the reaction temperature was 180 °C and the reaction time was 8 h, the I_R_/I_G_ ratio of the samples was about 10.6 times that when the reaction temperature was 180 °C and the reaction time was 4 h. As can be seen from Figure 5b, when the temperature was 180 °C and the reaction time was 8 h, the sample produced orange-upconversion luminescence and the calculated CIE color coordinates were located in the red region. As can be seen in Figure 5a, when the reaction temperature was 200 °C and the reaction time was 4 h or 8 h, the I_R_/I_G_ ratios of the samples were ~14.571 and ~15.106, respectively. The calculated CIE color coordinates almost overlapped and were located in the red area. These two samples produced red-upconversion luminescence, and the results can be seen in Figure 5b.

## 4. Conclusions

In summary, a series of Na_3_ScF_6_: 20% Yb^3+^, 2% Er^3+^ samples were synthesized by a solvothermal method. The samples prepared by adjusting the reaction time and temperature can produce green- and red-upconversion luminescence when excited by 980 nm near-infrared light. In addition, we also found that by controlling the size of the samples we could adjust the I_R_/I_G_ ratio. When the size of a sample was small, it exhibited green-upconversion luminescence, and when the size gradually increased, the upconversion luminescence gradually changed to red and the I_R_/I_G_ ratio also gradually increased. These results have great potential for research on color displays and lighting.

## Data Availability

Not applicable.
